# NGS‐based liquid biopsy profiling identifies mechanisms of resistance to ALK inhibitors: a step toward personalized NSCLC treatment

**DOI:** 10.1002/1878-0261.13033

**Published:** 2021-06-18

**Authors:** Estela Sánchez‐Herrero, Roberto Serna‐Blasco, Vadym Ivanchuk, Rosario García‐Campelo, Manuel Dómine Gómez, José M. Sánchez, Bartomeu Massutí, Noemi Reguart, Carlos Camps, Sandra Sanz‐Moreno, Silvia Calabuig‐Fariñas, Eloísa Jantus‐Lewintre, Magdalena Arnal, Dietmar Fernández‐Orth, Virginia Calvo, Víctor González‐Rumayor, Mariano Provencio, Atocha Romero

**Affiliations:** ^1^ Liquid Biopsy Laboratory Biomedical Sciences Research Institute Puerta de Hierro‐Majadahonda Spain; ^2^ Atrys Health Barcelona Spain; ^3^ Medical Oncology Department Complejo Hospitalario Universitario A Coruña Spain; ^4^ Medical Oncology Department Hospital Universitario Fundación Jiménez Díaz Oncohealth Institute Universidad Autónoma de Madrid Spain; ^5^ Medical Oncology Department Hospital La Princesa Madrid Spain; ^6^ Medical Oncology Department Hospital Universitario de Alicante ISABIAL Alicante Spain; ^7^ Medical Oncology Department Hospital Clinic of Barcelona Spain; ^8^ Molecular Oncology Laboratory Fundación Hospital General Universitario de Valencia Spain; ^9^ CIBERONC Valencia Spain; ^10^ Department of Medical Oncology Hospital General Universitario de Valencia Spain; ^11^ Department of Medicine Universitat de València Spain; ^12^ Department of Pathology Universitat de València Spain; ^13^ Department of Biotechnology Universitat de València Spain; ^14^ MARGenomics IMIM (Hospital del Mar Medical Research Institute) Barcelona Spain; ^15^ European Genome‐phenome Archive Centre for Genomic Regulation (CRG) Barcelona Spain; ^16^ Medical Oncology Department Hospital Universitario Puerta de Hierro‐Majadahonda Spain

**Keywords:** *ALK*‐TKI, *EML4‐ALK*, liquid biopsy, NGS, NSCLC

## Abstract

Despite impressive and durable responses, nonsmall cell lung cancer (NSCLC) patients treated with anaplastic lymphoma kinase (*ALK*) inhibitors (*ALK*‐Is) ultimately progress due to development of resistance. Here, we have evaluated the clinical utility of circulating tumor DNA (ctDNA) profiling by next‐generation sequencing (NGS) upon disease progression. We collected 26 plasma and two cerebrospinal fluid samples from 24 advanced *ALK*‐positive NSCLC patients at disease progression to an *ALK*‐I. These samples were analyzed by NGS and digital PCR. A tool to retrieve variants at the *ALK* locus was developed (V*ALK* tool). We identified at least one resistance mutation in the *ALK* locus in ten (38.5%) plasma samples; the G1269A and G1202R mutations were the most prevalent among patients progressing to first‐ and second‐generation *ALK*‐Is, respectively. Overall, 61 somatic mutations were detected in 14 genes: *TP53*, *ALK*, *PIK3CA*, *SMAD4*, *MAP2K1* *(MEK1)*, *FGFR2*, *FGFR3*, *BRAF*, *EGFR*, *IDH2*, *MYC*, *MET*, *CCND3,* and *CCND1*. Specifically, a deletion in exon 19 in *EGFR*, a non‐V600 *BRAF* mutation (G466V), and the F129L mutation in *MAP2K1* were identified in four patients who showed no objective survival benefit from *ALK*‐Is. Potential *ALK*‐I‐resistance mutations were also found in *PIK3CA* and *IDH2*. Finally, a c‐*MYC* gain, along with a loss of *CCND1* and *FGFR3*, was detected in a patient progressing on a first‐line treatment with crizotinib. We conclude that NGS analysis of liquid biopsies upon disease progression identified different putative *ALK*‐I‐resistance mutations in most cases and could be a valuable approach for therapy decision making.

## Graphical abstract

Despite impressive and durable responses, nonsmall cell lung cancer patients treated with *ALK* inhibitors (*ALK*‐Is) ultimately progress due to the development of resistance mechanisms. In this study, we show that next‐generation sequencing (NGS) analysis of liquid biopsies upon disease progression is feasible and informative as it can identify different mechanisms underlying resistance to *ALK*‐Is by NGS profiling of ctDNA.

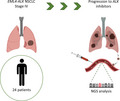

**Abbreviations**
AF: allele frequency
*ALK*
: anaplastic lymphoma kinase
*ALK*‐Is: Anaplastic lymphoma kinase inhibitors
cfDNA: cell‐free DNA
CNS: central nervous system
CNVs: copy‐number variations
CSF: cerebrospinal fluid
CTC: circulating tumor cell
dPCR: digital PCR
ECOG: cooperative oncology group
LOD: limit of detection
MAF: mutant allele frequency
MNP: multiple‐nucleotide polymorphism
NGS: next‐generation sequencing
NPA: negative percentage agreement
NSCLC: nonsmall cell lung cancer
ORA: overall rates of agreement
OS: overall survival
PFS: progression‐free survival
PPA: positive percentage agreement
SNVs: single nucleotide variants
UMIs: unique molecular identifiers
wt: wild‐type

## 1 Introduction

Anaplastic lymphoma kinase (*ALK*) inhibitors (*ALK*‐Is) have dramatically improved outcomes of nonsmall cell lung cancer (NSCLC) patients whose tumors harbor an *ALK* translocation [1, 2]. A broad therapeutic arsenal is currently available to treat *ALK*‐positive NSCLC tumors, and sequential treatment with different *ALK*‐Is is the best therapeutic option for NSCLC patients with an *ALK* translocation [3, 4]. However, it remains unclear how *ALK*‐Is should be sequenced. It has been proposed that treatment sequencing can be established according to clinical characteristics of the patients or toxicity profile. In this way, second‐generation *ALK*‐Is have shown impressive central nervous system (CNS) efficacy in *ALK*‐positive NSCLC patients [5, 6]. On the other hand, crizotinib is associated with adverse events dominated by gastrointestinal and visual effects, increased transaminases and edema, whereas the most common adverse events of alectinib are anemia, myalgia, and increased blood bilirubin [7]. The tumor molecular profile can also determine the treatment response. In this way, among patients treated with the *ALK*‐I lorlatinib, the *ALK* fusion variant 3 was associated with significantly longer progression‐free survival (PFS) than variant 1 [8]. Finally, several resistance mutations have been identified upon progression to an *ALK*‐I [9, 10]. While some of them confer resistance to specific *ALK*‐Is, others do not [11, 12, 13]. Paradoxically, therapy is seldom decided based on the tumor molecular profile upon disease progression, and *ALK*‐Is are usually prescribed empirically. Conversely, biomarker testing after treatment failure is routinely performed in *EGFR*‐positive NSCLC patients, as recommended by clinical guidelines [14].

Repeat tumor biopsy upon disease progression is not always feasible. Nevertheless, there is considerable evidence showing that genotyping cell‐free DNA (cfDNA) is a valid and significantly faster approach than genotyping solid biopsies. Next‐generation sequencing (NGS) enables the interrogation of a large number of mutations and can be used with liquid biopsies [15].

In this study, we have analyzed 26 plasma and two cerebrospinal fluid (CSF) samples, from 24 patients, collected upon disease progression while being treated with an *ALK*‐I, in order to determine the clinical utility of liquid biopsies for *ALK*‐Is sequencing. In addition, we provide a pipeline specifically designed to detect somatic mutations in the *ALK* domain. Finally, we evaluate the clinical significance of somatic alterations in genes other than *ALK*.

## 2 Methods

### 2.1 Study population

Between June 2015 and July 2019, 24 stage IV, *ALK*‐positive NSCLC patients progressing on an *ALK*‐I (the *ALK* cohort), were prospectively recruited from six hospitals across Spain. The study protocol was approved by the Hospital Puerta de Hierro Ethics Committee (internal code 79‐18) and was conducted in accordance with the precepts of the Code of Ethics of The World Medical Association (Declaration of Helsinki). All patients provided their appropriate written informed consent to participate in the study prior to enrollment. Briefly, patients who were 18+ years of age and with a pathologically confirmed diagnosis of stage IV NSCLC with an *EML4*‐*ALK* translocation were eligible for inclusion. The identification of *EML4‐ALK* rearrangement was carried out by different methodologies, according to each participant center (Table S1). Specifically the *ALK* testing was performed by immunohistochemistry (IHC) using the Ventana *ALK* (D5F3) CDx assay on a Ventana BenchMark XT automated slide‐processing system (Ventana Medical Systems, Tucson, AZ, USA) or the mAb *ALK* (5A4) (Novocastra™; Leica Biosystems, Newcastle Upon Tyne, UK); Fluorescence *In Situ* Hybridization (FISH) using Vysis LSI *ALK* Dual Color Break Apart FISH probe kit (Vysis, Downers Grove, IL, USA) or *ALK* (2P23) Break Apart FISH probe kit (cYTOtEST, Rockville, MD, USA). The nCounter analysis system (NanoString Technologies, Seattle, WA, USA) was also used to detect *EML4‐ALK* translocation. All plasma samples were collected upon disease progression, which was assessed according to RECIST criteria v.1.1. In total, 26 plasma and two CSF specimens were collected and analyzed.

### 2.2 Laboratory procedures

Peripheral whole‐blood samples were collected in a 10‐mL Streck cfDNA BCT® (Streck, Omaha, NE, USA) tube for cfDNA. CSF samples were collected in a 10‐mL sterile tube with no additives or anticoagulants. CSF samples were taken from patients progressing at the brain level. Samples were centrifuged at room temperature in two consecutive centrifugations of 1500 ***g*** for 10 min and 5000 ***g*** for 20 min in order to separate plasma or CSF from the cellular fraction. cfDNA was isolated using QIAamp Circulating Nucleic Acid Kit (QIAgen, Valencia, CA, USA) according to the manufacturer's instructions. Libraries were prepared from at least 15ng of input cfDNA using the Oncomine™ Pan‐Cancer Cell‐Free Assay kit (Thermo Fisher, Palo Alto, CA, USA) according to the manufacturer's instructions. According to manufacturer, this amplicon‐based targeted sequencing assay allows the detection of multiple variants in ctDNA isolated from liquid biopsy samples with a limit of detection (LOD) down to 0.1% mutant allele frequency (MAF). Specifically, the panel interrogates variants in 52 genes (Table S2). AMPureXP magnetic beads (Beckman Coulter, Inc., Brea, CA, USA) were used to purify all libraries. Subsequently, the individual libraries were quantified using the Ion Library TaqMan® Quantitation Kit (Thermo Fisher, Palo Alto, CA, USA) in a StepOnePlus™ qPCR machine (Thermo Fisher) and adjusted to a final concentration of 50 pm. Eight samples were pooled. Templating and Ion 550™ Chip loading were carried out with an Ion Chef™ System (Thermo Fisher). Finally, an Ion GeneStudio™ S5 Sequencer (Thermo Fisher) was used to sequence loaded Ion 550™ chips. torrent suite Software (v5.12) was used to analyze the raw sequencing data. The coverage analysis (v. 5.12.0.0) plugin was used for sequencing coverage analysis (Thermo Fisher). Raw reads were aligned to the human reference genome hg19. Variant calling, annotation, and filtering were performed on the Ion Reporter (v5.10, Thermo Fisher) platform using the Oncomine TagSeq Pan‐Cancer Liquid Biopsy workflow (v2.1, Thermo Fisher). All candidate mutations were manually reviewed using the Integrative Genomics Viewer v.2.3.40, (Broad Institute, Cambridge, MA, USA). The clinical significance of somatic variants was determined according to the Standards and Guidelines for the Interpretation and Reporting of Sequence Variants in Cancer [16].

The mutations identified by NGS were confirmed by digital PCR (dPCR) using a QuantStudio® 3D dPCR System (Applied Biosystems, South San Francisco, CA, USA). In accordance with the manufacturer's specifications, dPCR reactions were performed in an 18‐μL volume comprising 9 μL of 20X QuantStudio 3D Master Mix, 0.45 μL of 40× commercially available predesigned or custom TaqMan® assays and 8.55 μL of cfDNA (minimum amount 2 ng). Subsequently, 14.5 μL of the PCR reaction was loaded onto a QuantStudio 3D dPCR 20K chip using QuantStudio™ 3D dPCR Chip Loader. Each dPCR run included a negative control DNA, as a wild‐type (wt) control, a blank (with no cfDNA) and a positive control. PCR reactions were performed in a thermal cycler (Applied Biosystems) at 96 °C for 10 min, then 40 cycles at 56 °C for 2 min and 98 °C for 30 s, and a final elongation step at 72 °C for 10 min. Finally, samples were maintained at 22 °C for at least 30 min. Chips fluoresce was read twice two independent QuantStudio™ 3D dPCR Instruments. Results were visualized and analyzed using QuantStudio® 3D Analysis Suite™ Cloud Software (Thermo Fisher). The automatic call assignments for each data cluster were manually adjusted when needed. The MAF was calculated as the ratio of mutant DNA molecules to the sum of mutant and wt DNA molecules.

The LOD and limit of quantitation (LOQ) of the dPCR assays for *ALK* variants were evaluated for four custom TaqMan® assays by mixing DNA from two different plasmids an one patient sample harboring the specific *ALK* mutations in a background of wt DNA (from healthy donors) at different mutant allele concentrations (i.e., 1%, 0.5%, 0.1%, 0.05%). LOD and LOQ were estimated for an input of cfDNA of 7 ng. Specifically, LOD and LOQ for *ALK* G1202R assay were estimated using a patient sample. The mutation was previously confirmed by NGS. LOD and LOQ for *ALK* S1206Y were estimated using *ALK*_1 plasmid. Finally, LOD and LOQ for *ALK* L1196M and G1269A assays were estimated using *ALK*_3 plasmid. Plasmids were designed by Classic GeneArt Gene Synthesis Portal (Thermo Fisher). Schematic plasmid maps are available upon request. LOD and LOQ were calculated based on the standard deviation of the response and the slope according to ICH Q2 (R1) guidelines (Validation of analytical procedures: text and methodology). The standard deviation of the response was calculated based on standard error of the y‐intercept. Results of the detection sensitivity of TaqMan assays are displayed in Data S1. Overall, mutant allele frequencies correlated with the expected mutant allele frequencies in all cases (Pearson's correlation coefficient, 0.9999, 0.9991, 0.9994, and 0.9994 for G1202R, S1206Y, L1196M, and G1269A, respectively). LOD were 0.05%, 0.23%, 0.19%, and 0.18% for G1202R, S1206Y, L1196M, and G1269A, respectively. Additionally, 10 wt cfDNA from healthy donors were used to evaluate the false‐positive signals. *ALK* mutations were not detected in any of the wt samples.

Samples were considered to be positive when the MAF was greater than or equal to 0.1% and when there were at least 300 copies·mL^−1^ of wt DNA. A negative control DNA (wt) and a blank sample (containing no DNA) were included in every run.

In order to discard that mutations detected by NGS were not clonal hematopoiesis‐derived mutations (specifically those in *TP53*), DNA from peripheral blood mononuclear cells (PBMCs) was analyzed in 10 samples by dPCR or Sanger sequencing (Table S3). To this aim, DNA was isolated from PBMCs using Maxwell® RSC Whole Blood DNA Kit (Promega Corporation, Madison, WI, USA) according to the manufacturer's instructions. Custom primer sequences to target specific *TP53* regions (exon 2, exon 4, exon 7, and exon 8 splice site) were designed using the Primer3plus software. PCR reactions were performed in a 15‐μL volume comprising 7.5 μL of 20X QuantStudio 3D Master Mix, 5.5 μL of H_2_O, 0.5 μL of forward primer, 0.5 μL of reverse primer, and 1 μL of isolated DNA. PCR amplifications were carry out in the VeritiPro Thermal Cycler (Applied Biosystems). The thermal program included an initial step of 94 °C for 10 min, follow by 40 cycles of denaturation at 94 °C for 30 s, annealing at 60 °C for 1 min and extension at 72 °C for 30 s, and finally, at 72 °C for 7 min. Amplified DNA samples were subjected to electrophoresis with 2% agarose gel, stained with GelRed ™ Nucleic Acid Gel Stain, 10 000X in Water (Biotium, Hayward, CA, USA), and examined under an UV transilluminator. Products revealing clear PCR bands were purified by adding 2 µL of ExoSAP‐IT reagent (GE Healthcare®, Madison, WI, USA) for each 5 µL of preamplification volume and incubated for 15 min at 37 °C followed by 15 min at 80 °C. One microliter of PCR product was subjected to Sanger sequencing using PCR primers and the BigDye™ Terminator v1.1 Cycle Sequencing Kit (Applied Biosystems). The thermal program included an initial step of 94 °C for 10 min, follow by 25 cycles of denaturation at 94 °C for 10 s, annealing at 50 °C for 10 s and extension at 60 °C for 2 min. The PCR product was purified with the protocol of the BigDye® XTerminator™ Purification Kit (Applied Biosystems) and sequenced with the forward and reverse PCR primers on ABI PRISM 310 Genetic Analyzer (Applied Biosystems). Sequence data were analyzed on sequencing analysis Software 7 v7.0 (Applied Biosystems).

To increase the detection rate for variants at the *ALK* locus, we have developed a bioinformatic pipeline, called the V*ALK* tool (available at GitHub: https://github.com/AtochaHUPH/VALK‐tool‐), which is capable of fully automating the filtering generating a.csv file containing the output variants and a list of their properties. Specific conditions for single nucleotide variants (SNVs), indels, multiple‐nucleotide polymorphisms (MNP), fusions and copy‐number variation (CNV) calls were defined. The nonfiltered‐oncomine.tsv file with variants in ‘Variant Call Format' was obtained for the 28 samples. All variants that have passed the Oncomine Variants (v.5.12, Thermo Fisher) filter were included in the final analysis. In addition, we performed a second filtering process in order to rescue other somatic variants ruled out by the Oncomine Variants (v.5.12) filter. Specifically, parameters such as the overall error of the NGS assay, the LOD, the coverage depth, the percentage of targeted bases sequenced at that coverage depth, the total number of target reads covering a variant region, the number of reads supporting a specific variant, and the clinical significance, among others, were taken into account for the selection of the different thresholds. Figure S1 shows the selection criteria based on certain variables as presented in the nonfiltered‐oncomine.tsv file.

All computations were performed in r v.3.6.3 (R Foundation for Statistical Computing, Vienna, Austria) using additional packages. Specifically, the Tcl/Tk package that was used to provide the end‐user an intuitive graphical interface to carry out the entire filtering process. In addition, the Scales (v1.1.1; https://scales.r‐lib.org/) package, which contains functions that convert data values to perceptual properties, was used to transform the raw data obtained from the nonfiltered‐oncomine.tsv files into interpretable values for the end‐user. Positive and negative percentage agreement (PPA and NPA) and overall rates of agreement (ORA) of the V*ALK* tool for detecting the *ALK* mutations specified in Table S4 were calculated considering the imperfect reference standard the dPCR result and using the two independent data sets, the *ALK* cohort and the Valencia cohort, which consists of 54 cfDNA samples from NSCLC patients.

### 2.3 Statistical analysis

Median follow‐up was estimated by the reverse Kaplan–Meier method. Overall survival (OS) was defined as the time from the start of treatment with an *ALK* inhibitor to death or last follow‐up. PFS was defined as the time between the start of an *ALK* inhibitor and disease progression (as ascertained by RECIST criteria), death, or the censored date of the last assessment, whichever occurred first. The log‐rank test was used to assess statistical differences between Kaplan–Meier survival curves. Hazard ratios were estimated from the Cox model using a multivariable approach adjusted for sex, cooperative oncology group (ECOG) performance status, and lines of *ALK*‐TKI. Association between clinicopathological variables and genomic features was assessed by Mann–Whitney and Fisher's exact test as needed. Values of *P* < 0.05 were considered statistically significant. Statistical analyses were performed using Stata 15.1 (Stata Corporation, College Station, TX, USA).

## 3 Results

### 3.1 Study cohort

**Fig. 1:**
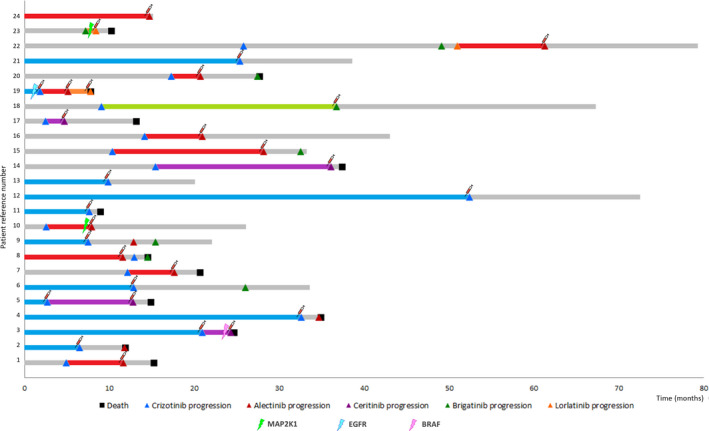
Swimmer chart showing the individual treatment responses of the study cohort. Blue, red, purple, green, and orange bars correspond to response duration of patients who were treated with crizotinib, alectinib, ceritinib, brigatinib, and lorlatinib, respectively, and from whom a blood sample was taken at the time of disease progression (syringe icon). Gray bars denote treatments responses to therapy for which samples at disease progression could not be analyzed (out of study). Tumor progression is denoted by triangle, and patient's death is denoted by a squared. Mutations in *BRAF, EGFR,* and *MAP2K1* are indicated by a lightning.

We collected and analyzed 26 plasma and two CSF specimens from 24 metastatic patients diagnosed with an *ALK*‐positive NSCLC who were progressing on an *ALK*‐I. Baseline clinicopathological characteristics of the study population (*N* = 24) are presented in Table 1. The median age at diagnosis was 53 years (range, 36–72 years) and 58.3% were females. The majority of the patients were never smokers (62.5%) and the most frequent histology was adenocarcinoma (95.8%). ECOG Performance Status at study entry varied from 0 to 2. As shown in Fig. 1, two samples from two patients were collected upon disease progression while on two consecutive lines of treatment with an *ALK*‐I; three samples were obtained from one patient upon failure to three consecutive lines of *ALK*‐I; all 21 other members of the cohort each provided a single sample.

**Table 1:** Baseline characteristics of the study cohort.

Feature		*N*	%
Age of diagnosis (years)	Median (range)	53 (36–72)	
Sex	Male	10	41.7
	Female	14	58.3
Smoking status	Current smoker	3	12.5
	Ex‐smoker	6	25
	Never‐smoker	15	62.5
ECOG performance status	0	12	50
	1	11	45.8
	2	1	4.2
Histology	Adenocarcinoma	23	95.8
	Neuroendocrine carcinoma	1	4.2
Clinical stage at diagnosis	III	6	25
	IV	18	75

As presented in Fig. 1, 13 samples corresponded to *ALK*‐I‐naïve patients who progressed on a first‐line crizotinib (*N* = 11) or alectinib (*N* = 2) treatment. For these patients, the median PFS and OS were 11.6 months (95% CI: 6.5–20.9 months) and 24.6 months (95% CI: 11.8–NR months), respectively. In addition, 12 samples corresponded to patients who had received previously crizotinib and were treated with a second‐generation *ALK*‐I. Finally, the cohort included two samples from patients progressing on lorlatinib after failure of a prior second‐generation *ALK*‐I and one patient progressing on alectinib who had previously received crizotinib plus two second‐generation *ALK*‐I. Detailed information about treatment lines is presented in Table S5. The median PFS and OS for patients progressing on a second or subsequent line with an *ALK*‐I were 5.4 months (95% CI: 2–9.1) and 11.2 months (95% CI: 3–NR) months, respectively.

### 3.2 Next‐generation sequencing analysis upon disease progression

Overall plasma samples yielded higher cfDNA concentrations than CSF. Despite the small sample size of the study cohort, the median concentration of cfDNA isolated from CSF was significantly lower (0.20 ng·μL^−1^) than median concentration of cfDNA obtained from plasma samples (1.94 ng·μL^−1^; *P* = 0.039; calculated by Mann–Whitney test).

The average mapped reads per sample was 9 775 623, resulting in a median overall sequencing depth of 25 322. The median read coverage per sample was 21 261 and the median molecular coverage per sample was 1670.8. Regarding the two CSF samples, taken from patients with CNS disease, the average mapped reads per sample was 8 268 194, resulting in a median overall sequencing depth of 9318.

**Fig. 2:**
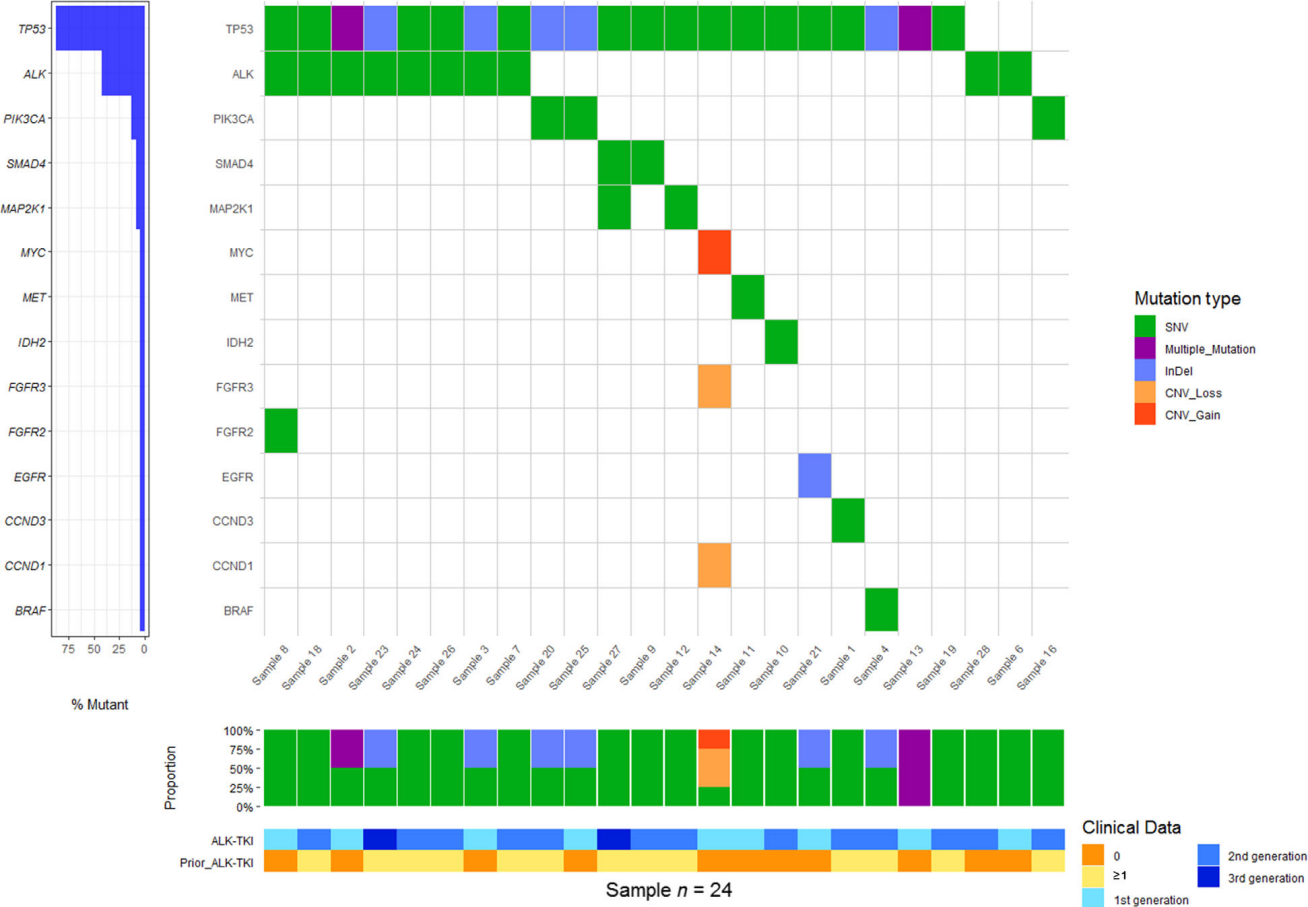
Co‐mutation plot according to ctDNA profiling by NGS. Each column represents data from a single patient. Each row represent data from a specific gene. Indels are represented in blue whereas missense mutations are represented in green. CNVs are colored in orange. Heat map at the bottom illustrates type of treatment received.

In total, 61 somatic variants in ctDNA from 24 samples were detected. In three patients, no mutations were found. One of the patients with undetectable plasma ctDNA had progressed exclusively at the brain level. The average number of mutations per patient was 2.18 and the median MAF was 0.39%, with a minimum MAF of 0.02% and a maximum of 5.09%. As expected, SNPs were the most frequent mutation type (*N* = 48). In addition, we identified 10 indels and three CNVs (Fig. 2). Specifically, a c‐*MYC* gain in conjunction with a *CCND1* and an *FGFR3* loss was detected in a patient progressing on a first line with crizotinib. This patient also harbored a mutation in *TP53* (Fig. 2).

As illustrated in Fig. 2, somatic mutations were detected in 14 genes: *TP53, ALK, PIK3CA, SMAD4, MAP2K1* *(MEK) FGFR2, FGFR3, BRAF, EGFR, IDH2, MYC, MET, CCND3, and CCND1*. All the variants detected are listed in Table S6. Thirteen variants (12 of which were in the *ALK* locus and one was in the *EGFR* gene) were categorized as being of strong clinical significance. Mutations in *TP53* gene were further tested in PBMCs in order to discard a clonal hematopoietic origin (Table S3).

### 3.3 Identification of acquired resistance mutations in the *ALK* locus upon disease progression

To increase the sensitivity for detecting somatic mutations in the *ALK* locus, we developed an algorithm named V*ALK* tool. The tool has been specifically designed for the analysis of the NGS data obtained from liquid biopsies. Among other parameters, the algorithm takes into account the molecular depth and molecular counts as well as specific regions that are more likely for false‐positive calls. In order to test the analytical performance of the tool, all SNPs in the *ALK* locus that were present in the nonfiltered‐oncomine.tsv file were analyzed by dPCR. In total, 19 *ALK* variants from 22 samples were evaluated (Table S4). Considering the nonreference standard the dPCR result PPA, NPA, and overall percent agreement of *ALK* mutation detection for the V*ALK* tool were 67% (95% CI: 35–90%), 93% (95% CI: 75–99%), and 85% (95% CI: 69–94%), respectively (Table S7). To validate the final algorithm (locked prior to analyses), a second independent batch of 54 samples provided by a different laboratory (Valencia cohort) was used. In this case, the PPA, NPA, and ORA were 100% (95% CI: 29–100%), 98% (95% CI: 90–100%), and 98% (95% CI: 90–100%), respectively. Cross‐tables describing PPA, NPA, and ORA for the V*ALK* tool as well as for the Oncomine variants v5.10 using *ALK* and Valencia cohorts are presented in Table S7–S10.

Overall, in the *ALK* cohort we detected at least one *ALK* mutation in 10 (38.5%) plasma samples collected upon disease progression (Table 2, and Fig. S2). Notably, the Oncomine variants v5.10 filter only detected *ALK* mutations in three patients (Table 2).

**Table 2:** Somatic mutations detected at the *ALK* locus upon treatment failure.

No. of patient	Nucleotide change	Amino acid change	Progression to	Line of treatment	Filter	MAF NGS (%)	MAF dPCR (%)	Total dPCR input (ng)
Patient 2	c.3599C>T	p.A1200V	Crizotinib	First line	nonfiltered‐oncomine.tsv	0.02	0.04	42.07
Patient 3	c.3806G>C	p.G1269A	Crizotinib	First line	Oncomine 5.10/V*ALK* tool	3.36	2.82	4.64
Patient 5	c.3806G>C	p.G1269A	Crizotinib	First line	Oncomine 5.10/V*ALK* tool	0.88	0.42	7.20
Patient 5	c.3604G>A	p.G1202R	Ceritinib	Second line	Oncomine 5.10/V*ALK* tool	1.28	2.12	54.04
Patient 6	c.3586C>A	p.L1196M	Crizotinib	First line	nonfiltered‐oncomine.tsv	0.02	0.06	27.10
Patient 16	c.3617C>A	p.S1206Y	Alectinib	Second line	V*ALK* tool	0.06	0.01	26.50
Patient 16	c.3604G>A	p.G1202R	Alectinib	Second line	V*ALK* tool	0.04	0.04	26.50
Patient 19	c.3586C>A	p.L1196M	Lorlatinib	Third line	nonfiltered‐oncomine.tsv	0.05	0.05	29.24
Patient 19	c.3824G>A	p.R1275Q	Lorlatinib	Third line	nonfiltered‐oncomine.tsv	0.03	0.04	21.80
Patient 20	c.3604G>A	p.G1202R	Alectinib	Second line	V*ALK* tool	0.05	0.32	14.11
Patient 22	c.3604G>A	p.G1202R	Alectinib	Fourth line	V*ALK* tool	0.03	0.01	48.05
Patient 24	c.3538G>C	p.V1180L	Alectinib	First line	nonfiltered‐oncomine.tsv	0.37	0.35	16.67

The G1202R mutation was identified in four patients who had progressed on alectinib (*N* = 3) and ceritinib (*N* = 1; patient number 5). Specifically, in case of the patient progression on ceritinib (patient 5), this mutation was not detected at treatment initiation confirming its role as a resistance mutation (Table S11). In addition, the S1206Y mutation was detected along with the G1202R mutation in one of the aforementioned alectinib‐progressing patients. This patient had been treated with crizotinib before initiating alectinib treatment. The low MAF of the S1206Y mutation suggests that it could be responsible for the previous crizotinib failure and it could have been decreased since alectinib therapy. In addition, the G1269A mutation was detected upon crizotinib failure in two cases and the L1196M mutation was identified after progression to crizotinib and lorlatinib. The latter was detected together with the R1275Q mutation in a patient diagnosed with an *ALK*‐positive neuroendocrine carcinoma (patient 19). Of note, neither the L1196M mutation nor the R1275Q mutation was detected in the pre‐treatment plasma sample from patient 19 (Table S11). Finally, the A1200V mutation (*N* = 1) was detected in a sample collected after crizotinib failure, and the V1180L mutation (*N* = 1) was detected in a patient progressing on a first‐line treatment with alectinib (Table 2).

### 3.4 Other molecular mechanisms underlying resistance to *ALK*‐I

A deletion in exon 19 of the *EGFR* gene, a non‐V600 *BRAF* mutation, and the F129L mutation in *MAP2K1* (MEK1) were identified in four patients who showed no objective survival benefit from *ALK*‐Is. None of these patients had a secondary mutation in *ALK* locus.

Notably, the patient harboring the E746_A750del mutation in the *EGFR* gene had a PFS time of 1.8 months under first‐line crizotinib treatment. The patient was subsequently treated with alectinib but tumor progression was assessed 3.1 months later prompting a switch of treatment to lorlatinib, but that also failed after 1.8 months, suggesting that the tumor had primary resistance to *ALK*‐Is (Fig. 1, Table 3). Similarly, a non‐V600 *BRAF* mutation, namely G466V, was identified in the CFS collected upon disease progression to ceritinib. While PFS with first‐line crizotinib was 21 months, disease progression was assessed within 3 months of starting second‐line ceritinib treatment (Fig. 1). Remarkably, the non‐V600 *BRAF* mutation was absent in the plasma sample collected before ceritinib initiation (Table S11). Likewise, two patients harboring the F129L mutation in *MAP2K1* (*MEK1*) obtained little benefit from second‐line *ALK*‐I (Fig. 1, Table 3). This mutation was detected upon disease progression to alectinib (patient 10) and lorlatinib (patient 23). In case of patient 10, we could confirm that this mutation was not present in the pre‐treatment plasma sample and tumor biopsy (Table S11). Noteworthy, the median PFS and OS for second‐line treatment for these patients were less than one month (0.97) and 3 months, respectively, whereas median the PFS and OS for patients progressing on a second or subsequent lines with an *ALK*‐I but without mutations in *MAP2K1* were 5.9 and 11.2 months (*P* log‐rank < 0.05 in both cases; Fig. S3).

**Table 3:** Resistance mutations detected in loci other than *ALK* upon tumor progression.

Patient	Treatment	Treatment line	Sample	HUGO symbol	Amino acid change	Nucleotide change/CNV	Type	Variant class (Tier)	Transcript	rs	COSM
Patient 8	Alectinib	1st	Sample 10	*IDH2*	p.R140Q	c.419G>A	SNV	Potential clinical significance	NM_002168.3	–	COSM41590
Patient 14	Ceritinib	2nd	Sample 16	*PIK3CA*	p.E545K	c.1633G>A	SNV	Potential clinical significance	NM_006218	rs104886003	COSM763
Patient 18	Brigatinib	2nd	Sample 20	*PIK3CA*	p.E545A	c.1634A>C	SNV	Potential clinical significance	NM_006218	rs121913274	COSM12458
Patient 3	Ceritinib	2nd	Sample 4	*BRAF*	p.G466V	c.1397G>T	SNV	Potential clinical significance	NM_004333.4	rs121913351	COSM451
Patient 10	Alectinib	2nd	Sample 12	*MAP2K1*	p.F129L	c.385T>C	SNV	Potential clinical significance	NM_002755.3	rs1057519805	COSM1570285
Patient 12	Crizotinib	1st	Sample 14	*MYC*		Gain (3.08)	CNV	Potential clinical significance	NM_005359.5	–	
Patient 19	Crizotinib	1st	Sample 21	*EGFR*	p.E746_A750del	c.2235_2249delGGAATTAAGAGAAGC	InDel	Strong clinical significance	NM_005228.4	–	COSM6223
Patient 23	Lorlatinib	2nd	Sample 27	*MAP2K1*	p.F129L	c.385T>C	SNV	Potential clinical significance	NM_002755.3	rs1057519805	COSM1570285

Potential *ALK*‐I resistance mutations were also found in *IDH2, PIK3CA,* and *MYC* (Table 3). Specifically, the oncogenic mutations E545K and E545A in the *PIK3CA* gene were detected in the plasma sample of two patients progressing on ceritinib and brigatinib (Table 3). These mutations were not detected in the pre‐treatment sample (Table S11). Likewise, the gain‐of‐function mutation in *IDH2*, R140Q, was detected upon disease progression to first‐line alectinib treatment. Finally, as previously mentioned, a c‐*MYC* amplification was detected jointly with a loss of *CCND1* and of *FGFR3*.

## 4 Discussion

*ALK*‐Is have dramatically improved outcomes in NSCLC patients [17]. However, despite the impressive responses they elicit, patients invariably relapse due to acquired resistance mutations. Solid biopsies remain the gold standard for biomarker testing. However, logistics for obtaining repeat tumor biopsies are complicated and seldom feasible leading to an empirical prescription of sequential *ALK*‐Is. Nevertheless, blinding treatment sequential strategies might have a deleterious effect on patient's survival due to the incompletely overlapping *ALK* mutation coverage of different *ALK*‐Is. In this exploratory analysis, we show that plasma NGS is feasible and we propose several different mechanisms which may underlie resistance to *ALK* Inhibitors (*ALK*‐Is). Unfortunately, we did not have available data or plasma/tissue samples collected at baseline in 18 of the 24 patients included in the study, which may constitute an important limitation. Importantly, we also provide an algorithm capable of retrieving somatic mutations in the *ALK* locus that would otherwise be discarded by the commercial bioinformatic pipeline. As presented in Table 2, the commercial pipeline only detected three out of 12 mutations. MAFs of variants detected by the commercial pipeline were 2.8%, 2.1%, and 0.4%. According to the manufacturer's specifications, the LOD, in terms of MAF, for mutations is 0.1%. However, in our hands, mutations with a MAF below 0.5% are seldom detected by the commercial pipeline. By using the V*ALK* pipeline, some mutations that would otherwise have been missed can be rescued. Yet, confirmation using an alternative technique such as dPCR would be required to rule out false‐positive calls.

Regarding acquired mutations in the *ALK* locus, our results are consistent with those of previous studies. Specifically, secondary mutations were detected in the plasma samples of 4 of the 11 (36%) patients treated with first‐line crizotinib, with the G1269A mutation being detected in two cases. In this regard, mutation detection rate after crizotinib failure might vary from 60% [18] to 24% [19], G1269A being the most prevalent mutation. We also detected the L1196M and S1206Y mutations, which have been reported to occur in 7% and 2% of cases, respectively, of *ALK*‐positive NSCLC patients treated with crizotinib [11]. Finally, we detected the A1200V mutation after crizotinib failure in one patient. This mutation is also known to appear upon crizotinib progression [18]. In addition, we found that the G1202R mutation was identified in three of the 10 patients (30%) progressing on alectinib. This mutation is known to arise after treatment with second‐generation *ALK*‐Is [11]. Recently, Noé *et al*. [20] reported a 53% *ALK* mutation detection rate in samples obtained post‐progression on alectinib in which G1202R was the most frequent mutation. In our cohort, more than one mutation in *ALK* locus was detected in two samples collected during second‐ and third‐generation *ALK*‐I treatment. Likewise, it has been described that *ALK* resistance mutations become more frequent with each successive generation of *ALK*‐I as sequential treatment may promote the appearance of resistance mutation at the *ALK* locus [21].

A reduced number of studies analyzing samples collected upon progression to an *ALK*‐I by NGS have so far been conducted [11, 18]. To our knowledge, there are only two studies describing NGS analysis of *EML4‐ALK* NSCLC liquid biopsy samples using Oncomine™ Pan‐Cancer Cell‐Free Assay and both are case reports [22, 23]. Overall all, our results are in agreement with other studies [11]. However, some genes such as *IGF‐1R* and *SRC,* which have been described to be relevant for *ALK* resistance, were not included in the used panel. Genomic alterations responsible of treatment failure can be detected analyzing different components of bloodstream, most notably circulating tumor cells (CTCs) and cfDNA [19, 24, 25]. However, it has been proposed that cfDNA is the best strategy for sequencing analyses [26]. Nonetheless, assays for the detection of *EML4‐ALK* fusion protein in CTCs have been developed [27]. Consistent with our results, mutations in *TP53, FGFR2, PIK3CA, and MET* have been identified in the tumor biopsy of patients progressing on ceritinib [11]. The E545K and E545A mutations in *PIK3CA* have not only been detected upon progression in advanced *ALK*‐positive NSCLC patients, but also in *EGFR‐*positive NSCLC patients [28, 29]. The *IDH2* R140Q detected in our cohort is known to transform cells in vitro and induces myeloid and lymphoid neoplasms in mice [30, 31]. This mutation is also frequent in angioimmunoblastic T‐cell lymphoma [32]. In NSCLC, *IDH1/2* mutations are rarely detected in primary tumors but it has been suggested that they could be branching drivers leading to subclonal evolution, based on the MAFs at which these mutations are detected [33]. In this way, Zhao *et al*. [34] described a case of *an ALK‐*positive tumor in which an *IDH1* variant was detected upon disease progression.

Also, we found the E746_A750del mutation in one patient who did not benefit from treatment with *ALK*‐Is. Unfortunately, we could not orthogonally validate the presence of the *ALK* translocation nor the *EGFR* variant at baseline due to lack of tissue, which may constitute a limitation of the present study. However, it has been previously reported that mutations in *EGFR* in some NSCLC tumors coexist alongside *ALK* rearrangements, although this is, at best, a rare event [35, 36]. Concomitant *ALK* and *EGFR* alterations may lead to primary resistance to *ALK*‐I [37]. Likewise, a non‐V600 *BRAF* mutation was detected after 3 months of treatment with second‐line ceritinib treatment, suggesting that resistance of the tumor to the *ALK*‐I could be due to the acquisition of the *BRAF* mutation. It has been reported that ceritinib enhances the efficacy of trametinib, a MEK inhibitor, in *BRAF/NRAS*‐wt melanoma cell lines [38], which makes it plausible that ceritinib would not have any effect in *BRAF*‐mutated cells. Finally, two patients in whose plasma sample the F129L‐activating mutation in *MAP2K1* (MEK1) was detected, exhibited marked resistance to second‐ and third‐generation *ALK*‐Is. Of note, we did not detect this mutation at baseline in one of these patients, supporting its role as a resistance mutation. In this way, this mutation has been identified as the molecular mechanism underlying *MEK/ERK* pathway activation in resistant clones of human HT‐29 colon cancer cells [39]. Moreover, the activation of this downstream pathway is critical to the survival of *ALK*‐positive NSCLC cells [40, 41]. Indeed, the combination of *ALK* and *MEK* inhibition was highly effective at suppressing tumor growth in a preclinical model of *EML4‐ALK* NSCLC [42]. Taken together, it is plausible that the F129L‐activating mutation in *MAP2K1* is an acquired mutation that leads to tumor resistance to *ALK*‐Is.

Mutations in the *FGFR2* and *FGFR3* genes were detected in two patients progressing on *ALK*‐Is, suggesting sensitivity to fibroblast growth factor receptor inhibitors. It has been reported that alectinib, despite being a potent *ALK*‐I, has limited inhibitory activity against other protein kinases such as *FGFR2* [43]. In this way, clinical trials evaluating the efficacy of combinations of *ALK*‐Is with *FGFR* inhibitors would be of particular interest.

Three CNVs in c‐*MYC*, *CCND1,* and *FGFR3* were detected upon disease progression in one patient, who was being treated with crizotinib. Remarkably, c‐*MYC* amplification determines many oncogenic effects [44] and it has been identified as a potential mechanism of primary resistance to crizotinib in *ALK*‐rearranged NSCLC patients [45]. It has been previously suggested by Alidousty *et al*. that co‐occurrence of early *TP53* mutations in *ALK* ^+^ NSCLC can lead to chromosomal instability. Specifically, authors reported that, in a subset of 53 *ALK* ^+^ tumors, up to a quarter of *TP53*‐mutated tumors showed amplifications of known cancer genes such as *MYC* or *CCND1* [46]. Consistent with this, we detected the P92A and V157F mutations in the *TP53* gene in the same plasma sample of this patient.

## 5 Conclusions

In conclusion, our data show that molecular mechanisms underlying treatment failure seem to involve different pathways. NGS analysis of liquid biopsies collected upon disease progression is feasible and a valuable approach toward personalized that will lead to better care for *ALK*‐positive NSCLC patients.

## Conflict of interest

MP reports personal fees from Roche, BMS, MSD Pfizer, Lilly, Novartis, and Takeda grants and personal fees from AstraZeneca, and Boehringer during the conduct of the study. VC reports personal fees from Roche BMS, MSD, Pfizer, Lilly, AstraZeneca, Boehringer, Novartis, Takeda, during the conduct of the study. MD reports personal fees from Astra‐Zeneca, BMS, Boehringer Ingelheim, MSD, Pfizer and Roche. The rest of the authors have declared no conflict of interest.

## Author contributions

AR and MP conceived and /or designed the work. ES and RS have carried out statistical analyses. VI has developed V*ALK* tool. All authors have made substantial contributions to the acquisition and interpretation of data. AR and ES have drafted and revised the manuscript. All authors have approved the final version. Each author agreed to be accountable for all aspects of the work in ensuring that questions related to the accuracy or integrity of any part of the work are appropriately investigated and resolved.

### Peer Review

The peer review history for this article is available at https://publons.com/publon/10.1002/1878‐0261.13033.

## Supporting information

**Fig. S1**. Flowchart of the bioinformatic pipeline optimized for the processing and assessment of variants at the *ALK* gene locus.
**Fig. S2**. Frequency of *ALK* missense mutations identified in the study population.
**Fig. S3**. PFS and OS curves according to F129L (*MAP2K1*) mutation status.
**Table S1**. Identification of *EML4‐ALK* translocation.
**Table S2**. Genes included in the NGS panel used.
**Table S3**. DNA genotyping of PBMCs.
**Table S4**. List of mutations in *ALK* locus tested by dPCR.
**Table S5**. *ALK*‐Is treatments of the study cohort.
**Table S6**. List of all somatic mutations detected in the study cohort.
**Table S7**. Cross‐table describing PPA, NPA and ORA for *ALK* cohort using V*ALK* tool.
**Table S8**. Cross‐tables describing PPA, NPA and ORA for Valencia cohort using V*ALK* tool.
**Table S9**. Cross‐tables describing PPA, NPA and ORA for *ALK* cohort using the Oncomine Filter.
**Table S10**. Cross‐tables describing PPA, NPA and ORA for Valencia cohort using the Oncomine Filter.
**Table S11**. List of the mutations detected at disease progression and status at baseline or in previous sample.

## Data Availability

This paper is available as a preprint in Research Square server, https://doi.org/10.21203/rs.3.rs‐86055/v1 (https://www.researchsquare.com/article/rs‐86055/v1).
