# Supplementary material for: NGS‐based liquid biopsy profiling identifies mechanisms of resistance to ALK inhibitors: a step toward personalized NSCLC treatment
Source: Mol Oncol. 2021 Jun 18;15(9):2363–76. doi: 10.1002/1878-0261.13033 (PMC8410554; doi:10.1002/1878-0261.13033)
Supplement: Supplementary file 1 — Fig. S1. Flowchart of the bioinformatic pipeline optimized for the processing and assessment of variants at the ALK gene locus. Fig. S2. Frequency of ALK missense mutations identified in the study population. Fig. S3. PFS and OS curves according to F129L (MAP2K1) mutation status. Table S1. Identification of EML4‐ALK translocation. Table S2. Genes included in the NGS panel used. Table S3. DNA genotyping of PBMCs. Table S4. List of mutations in ALK locus tested by dPCR. Table S5. ALK‐Is treatments of the study cohort. Table S6. List of all somatic mutations detected in the study cohort. Table S7. Cross‐table describing PPA, NPA and ORA for ALK cohort using VALK tool. Table S8. Cross‐tables describing PPA, NPA and ORA for Valencia cohort using VALK tool. Table S9. Cross‐tables describing PPA, NPA and ORA for ALK cohort using the Oncomine Filter. Table S10. Cross‐tables describing PPA, NPA and ORA for Valencia cohort using the Oncomine Filter. Table S11. List of the mutations detected at disease progression and status at baseline or in previous sample. [file MOL2-15-2363-s001.docx]

1. **Supplemental Data**

**Limit of detection (LOD) and limit of quantitation (LOQ) for dPCR *ALK* variants custom TaqMan® assays**

**G1202R**

| Summary Output |  |  |  |  |  |  |
| --- | --- | --- | --- | --- | --- | --- |
|  |  |  |  |  |  |  |
| *Regresion Statistics* | |  |  |  |  |  |
| Multiple R | 0.999974519 |  |  |  |  |  |
| R Square | 0.999949038 |  |  |  |  |  |
| Adjusted R Square | 0.999936297 |  |  |  |  |  |
| Standard Error | 0.032364871 |  |  |  |  |  |
| Observations | 6 |  |  |  |  |  |
|  |  |  |  |  |  |  |
| ANOVA |  |  |  |  |  |  |
|  | *df* | *SS* | *MS* | *F* | *Significance F* |  |
| Regression | 1 | 82.21234139 | 82.21234139 | 78485.4679 | 9.73948E-10 |  |
| Residual | 4 | 0.00418994 | 0.001047485 |  |  |  |
| Total | 5 | 82.21653133 |  |  |  |  |
|  |  |  |  |  |  |  |
|  | *Coefficients* | *Standard Error* | *t Stat* | *P-value* | *Lower 95%* | *Upper 95%* |
| Intercept | -0.01540959 | 0.016593234 | -0.92866704 | 0.40562786 | -0.061479794 | 0.030660614 |
| X Variable | 101.3360813 | 0.361717466 | 280.1525797 | 9.7395E-10 | 100.3317926 | 102.34037 |
|  |  |  |  |  |  |  |
| LOQ | 0.16% |  |  |  |  |  |
| LOD | 0.05% |  |  |  |  |  |
|  |  |  |  |  |  |  |
| Residual Output |  |  |  |  |  |  |
| *Observation* | *Predicted Y* | *Residuals* |  |  |  |  |
| 1 | 10.11819854 | -0.016198542 |  |  |  |  |
| 2 | 5.051394476 | 0.035605524 |  |  |  |  |
| 3 | 0.997951223 | -0.017951223 |  |  |  |  |
| 4 | 0.491270817 | 0.000729183 |  |  |  |  |
| 5 | 0.085926491 | 0.033073509 |  |  |  |  |
| 6 | 0.035258451 | -0.035258451 |  |  |  |  |

**S1206Y**

| Summary Output |  |  |  |  |  |  |
| --- | --- | --- | --- | --- | --- | --- |
|  |  |  |  |  |  |  |
| *Regresion Statistics* | |  |  |  |  |  |
| Multiple R | 0.99954393 |  |  |  |  |  |
| R Square | 0.99908807 |  |  |  |  |  |
| Adjusted R Square | 0.99886008 |  |  |  |  |  |
| Standard Error | 0.13693202 |  |  |  |  |  |
| Observations | 6 |  |  |  |  |  |
|  |  |  |  |  |  |  |
| ANOVA |  |  |  |  |  |  |
|  | *df* | *SS* | *MS* | *F* | *Significance F* |  |
| Regression | 1 | 82.1694818 | 82.1694818 | 4382.28387 | 3.1195E-07 |  |
| Residual | 4 | 0.07500151 | 0.01875038 |  |  |  |
| Total | 5 | 82.2444833 |  |  |  |  |
|  |  |  |  |  |  |  |
|  | *Coefficients* | *Standard Error* | *t Stat* | *P-value* | *Lower 95%* | *Upper 95%* |
| Intercept | 0.13032351 | 0.07020405 | 1.85635311 | 0.13697766 | -0.06459419 | 0.32524121 |
| X Variable | 101.309663 | 1.53038471 | 66.1988208 | 3.1195E-07 | 97.0606341 | 105.558692 |
|  |  |  |  |  |  |  |
| LOQ | 0.69% |  |  |  |  |  |
| LOD | 0.23% |  |  |  |  |  |
|  |  |  |  |  |  |  |
| Residual Output |  |  |  |  |  |  |
| *Observation* | *Predicted Y* | *Residuals* |  |  |  |  |
| 1 | 10.2612898 | -0.04128983 |  |  |  |  |
| 2 | 5.19580667 | 0.05419333 |  |  |  |  |
| 3 | 1.14342014 | 0.07657986 |  |  |  |  |
| 4 | 0.63687183 | 0.16312817 |  |  |  |  |
| 5 | 0.23163318 | -0.07163318 |  |  |  |  |
| 6 | 0.18097834 | -0.18097834 |  |  |  |  |

**L1196M**

| Summary Output |  |  |  |  |  |  |
| --- | --- | --- | --- | --- | --- | --- |
|  |  |  |  |  |  |  |
| *Regresion Statistics* | |  |  |  |  |  |
| Multiple R | 0.99969521 |  |  |  |  |  |
| R Square | 0.99939051 |  |  |  |  |  |
| Adjusted R Square | 0.99923813 |  |  |  |  |  |
| Standard Error | 0.11112779 |  |  |  |  |  |
| Observations | 6 |  |  |  |  |  |
|  |  |  |  |  |  |  |
| ANOVA |  |  |  |  |  |  |
|  | *df* | *SS* | *MS* | *F* | *Significance F* |  |
| Regression | 1 | 80.9974858 | 80.9974858 | 6558.82716 | 1.3933E-07 |  |
| Residual | 4 | 0.04939754 | 0.01234939 |  |  |  |
| Total | 5 | 81.0468833 |  |  |  |  |
|  |  |  |  |  |  |  |
|  | *Coefficients* | *Standard Error* | *t Stat* | *P-value* | *Lower 95%* | *Upper 95%* |
| Intercept | -0.03955517 | 0.05697441 | -0.69426204 | 0.52573106 | -0.19774149 | 0.11863115 |
| X Variable | 100.584571 | 1.2419905 | 80.9865863 | 1.3933E-07 | 97.1362523 | 104.032889 |
|  |  |  |  |  |  |  |
| LOQ | 0.57% |  |  |  |  |  |
| LOD | 0.19% |  |  |  |  |  |
|  |  |  |  |  |  |  |
| Residual Output |  |  |  |  |  |  |
| *Observation* | *Predicted Y* | *Residuals* |  |  |  |  |
| 1 | 10.0189019 | 0.0910981 |  |  |  |  |
| 2 | 4.98967336 | -0.18967336 |  |  |  |  |
| 3 | 0.96629054 | 0.01370946 |  |  |  |  |
| 4 | 0.46336768 | 0.03663232 |  |  |  |  |
| 5 | 0.0610294 | 0.0589706 |  |  |  |  |
| 6 | 0.01073711 | -0.01073711 |  |  |  |  |
|  |  |  |  |  |  |  |
|  |  |  |  |  |  |  |

**G1269A**

| Summary Output |  |  |  |  |  |  |
| --- | --- | --- | --- | --- | --- | --- |
|  |  |  |  |  |  |  |
| *Regresion Statistics* | |  |  |  |  |  |
| Multiple R | 0.99971419 |  |  |  |  |  |
| R Square | 0.99942847 |  |  |  |  |  |
| Adjusted R Square | 0.99928558 |  |  |  |  |  |
| Standard Error | 0.10866516 |  |  |  |  |  |
| Observations | 6 |  |  |  |  |  |
|  |  |  |  |  |  |  |
| ANOVA |  |  |  |  |  |  |
|  | *df* | *SS* | *MS* | *F* | *Significance F* |  |
| Regression | 1 | 82.5945175 | 82.5945175 | 6994.72402 | 1.2252E-07 |  |
| Residual | 4 | 0.04723247 | 0.01180812 |  |  |  |
| Total | 5 | 82.64175 |  |  |  |  |
|  |  |  |  |  |  |  |
|  | *Coefficients* | *Standard Error* | *t Stat* | *P-value* | *Lower 95%* | *Upper 95%* |
| Intercept | -0.03360485 | 0.05571184 | -0.60319053 | 0.57891416 | -0.18828571 | 0.12107601 |
| X Variable | 101.571346 | 1.21446755 | 83.6344667 | 1.2252E-07 | 98.1994436 | 104.943249 |
|  |  |  |  |  |  |  |
| LOQ | 0.55% |  |  |  |  |  |
| LOD | 0.18% |  |  |  |  |  |
|  |  |  |  |  |  |  |
| Residual Output |  |  |  |  |  |  |
| *Observation* | *Predicted Y* | *Residuals* |  |  |  |  |
| 1 | 10.1235298 | 0.08647025 |  |  |  |  |
| 2 | 5.04496245 | -0.18496245 |  |  |  |  |
| 3 | 0.98210861 | 0.04789139 |  |  |  |  |
| 4 | 0.47425188 | 0.01574812 |  |  |  |  |
| 5 | 0.06796649 | 0.05203351 |  |  |  |  |
| 6 | 0.01718082 | -0.01718082 |  |  |  |  |

1. **Supplementary Figures.**


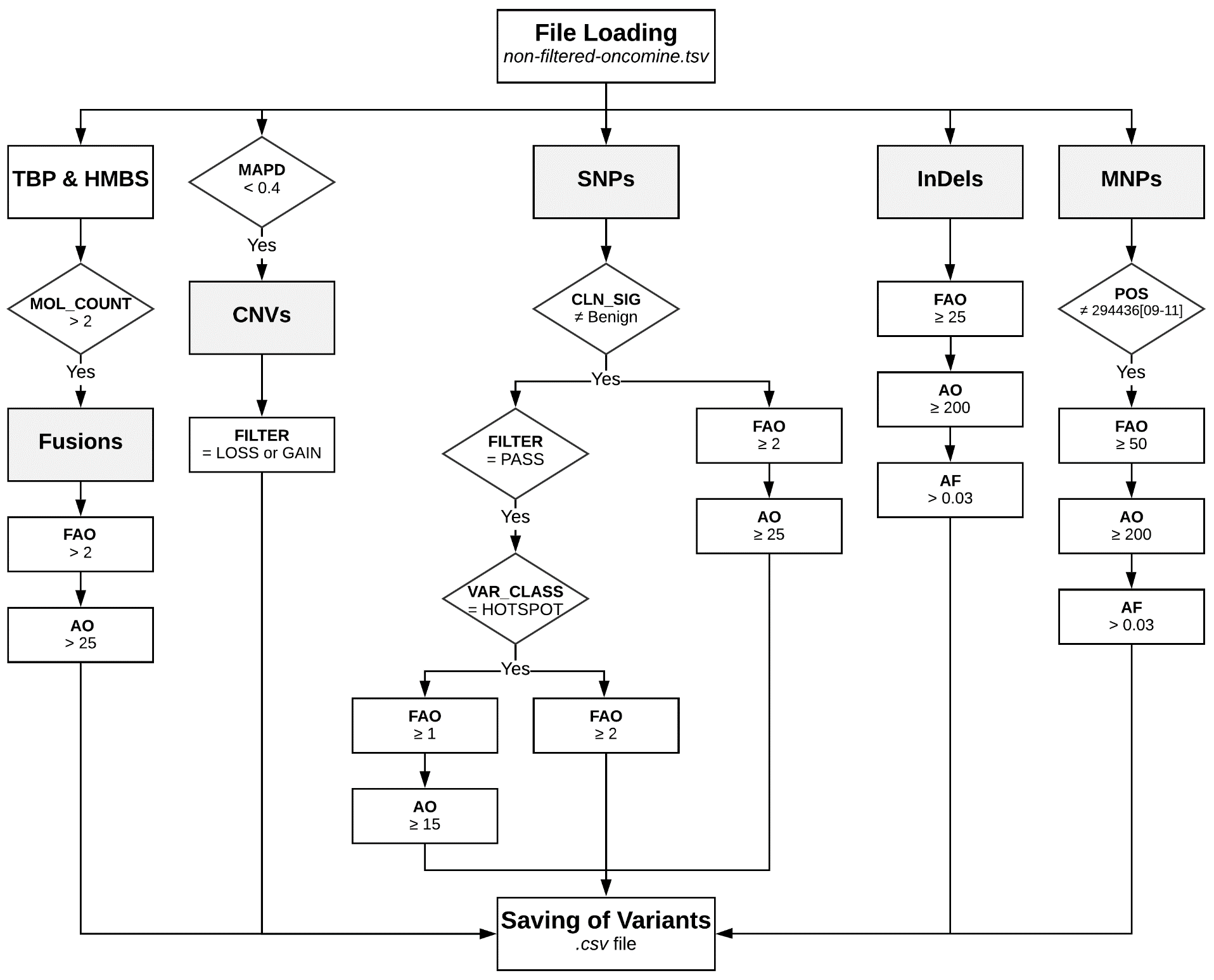


**Figure S1.** Flowchart of the bioinformatic pipeline optimized for the processing and assessment of variants at the *ALK* gene locus. FAO, molecular coverage; AO, read coverage; MAPD, median of the absolute values of all pairwise differences; FILTER: Ion Reporter™ internal filter (Oncomine™ Variants v5.12); CLN_SIG, clinical significance; VAR_CLASS: Oncomine™ Variant Class; POS: variant position; AF, allele frequency. **V*ALK* tool** selects fusion variants involving the *ALK* locus with molecular coverage >2 (INFO_MOL_COUNT) and fusion reads > 25 (INFO_READ_COUNT). The panel includes two control target genes, namely TBP and HMBS. Both controls must have a molecular count of >2 to pass QC (ROW_TYPE= “ProcControl”; FILTER= “PASS”). In addition, the tool selects *ALK* gene copy-number gains or losses (ROW_TYPE= “CNV”; FILTER= “GAIN” or FILTER= “LOSS”) when CNV Ratio is >1.15 or >0.85, respectively. As recommended by the manufacturer, to make a CNV call the P-value must be >10^-5^ and the MAPD (Median of the Absolute values of all Pairwise Differences) must be <0.4. MAPD is a quality metric that estimates coverage variability between adjacent amplicons in CNV analyses. The higher MAPD the lower coverage uniformity, resulting in a higher probability of erroneous CNV calls. Finally, according to our data false positives are less likely in SNPs and very likely in MNPs. For this reason, the algorithm makes a positive call as long as any of the following five conditions is met:

1. SNPs in the HotSpot file that have passed the Oncomine Variants 5.10 filter and that were detected in at least one molecular count with ≥15 reads (Rowtype= “SNP”; INFO.A.FAO ≥1; INFO.A.AO ≥15; FILTER= “PASS”; FUNC1.oncomineVariantClass= "Hotspot");

2. SNPs that have passed the Oncomine Variants (5.10) filter and that were detected in at least two molecular counts (Rowtype= “SNP”; INFO.A.FAO ≥2; FILTER= “PASS”; FUNC1.oncomineVariantClass= "Hotspot”);

3. SNPs that have been detected in at least two molecular counts with ≥25 reads (Rowtype= “SNP”; INFO.A.FAO ≥2; INFO.A.AO ≥25);

4. Variants different from MNPs that have been detected in ≥25 molecular counts with ≥200 reads and AF ≥0.03 (Rowtype≠ “mnp”; INFO.A.FAO ≥25; INFO.A.AO ≥200; INFO.A.AF >0.03)

5. All variants that have been detected in ≥50 molecular counts with ≥200 reads and an AF ≥0.03, excluding the positions chr2: 29443609 to 29443611 in the ALK locus, which involves six consecutive guanines (G) (INFO.A.FAO ≥50; INFO.A.AO ≥200: INFO.A.AF >0.03; POS≠ “29443609”, “29443610”, “29443611”).


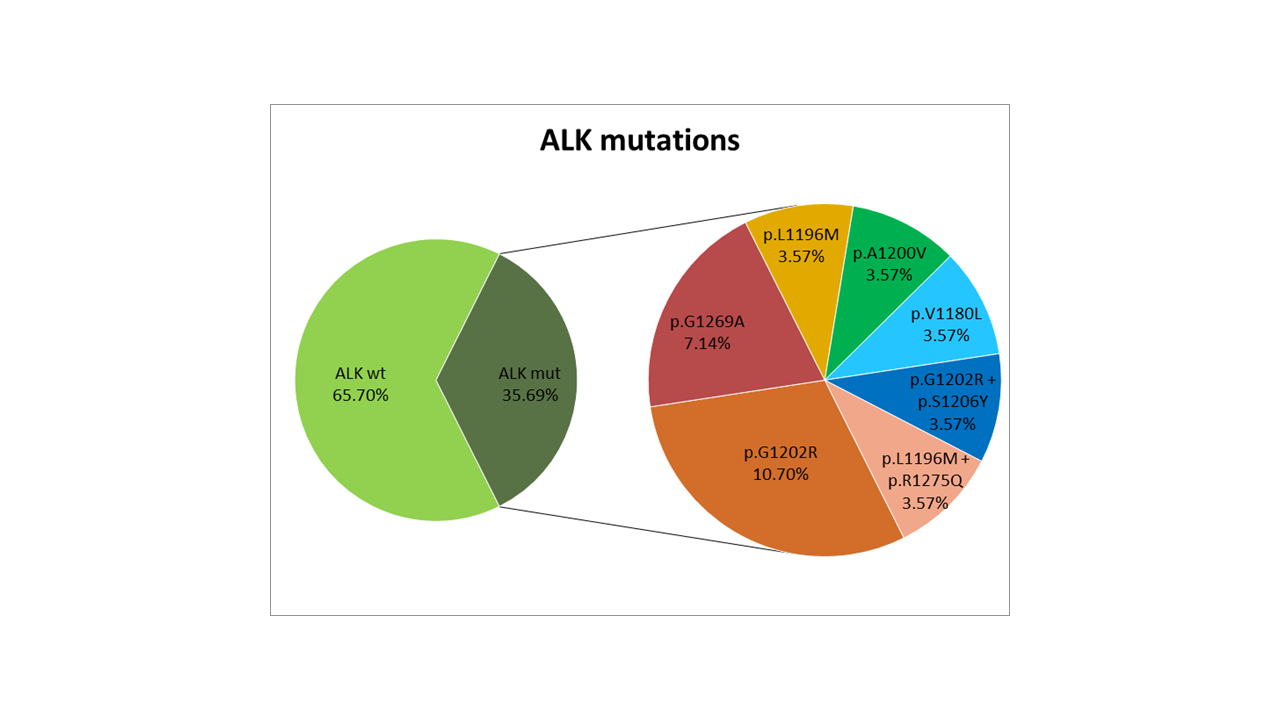


**Figure S2.** Frequency of *ALK* missense mutations identified in the study population


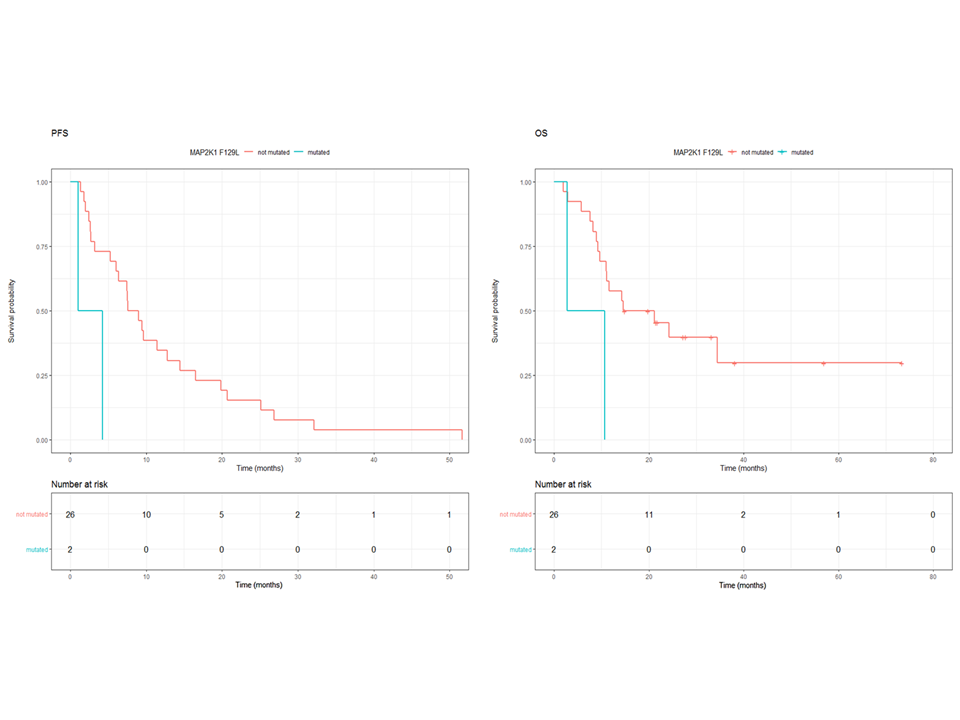


**Figure S3.** Progression free survival (PFS) and overall survival (OS) curves according to F129L (*MAP2K1*) mutation status. According to available clinical data there were not any clinicopathological feature that could explain the poor prognosis of F129L-positive cases (ECOG performance status was 1 in both cases, histology was adenocarcinoma in both positive cases and none of the F129L patients had brain metastases)

1. **Supplementary Tables.**

| **CENTER** | **IHC – *ALK* D5F3** | **IHC – *ALK* 5A4** | **FISH - Vysis LSI *ALK*** | **FISH - *ALK* (2P23)** | **nCounter** |
| --- | --- | --- | --- | --- | --- |
| HUPH |  |  | X |  |  |
| CHUAC | X |  |  |  |  |
| FJD | X |  | X (confirmatory) |  |  |
| HGUV |  | X |  | X (confirmatory) |  |
| HClinic | X |  |  |  | X (confirmatory) |
| HUA | X |  | X (confirmatory) |  |  |

**Table S1. Identification of *EML4-ALK* translocation.** Methodology used by the Pathology Department of the six participant hospitals for *EML4-ALK* testing.

HUPH - Hospital Universitario Puerta de Hierro-Majadahonda

CHUAC - Complejo Hospitalario Universitario A Coruña

FJD - Hospital Universitario Fundación Jiménez Díaz

HGUV - Fundación Hospital General Universitario de Valencia

HClinic - Hospital Clinic of Barcelona

HUA - Hospital Universitario de Alicante

| **GENES IN TARGET PANEL** | **bp INTERROGATED** | **GENES IN TARGET PANEL** | **bp INTERROGATED** |
| --- | --- | --- | --- |
| *AKT1* | *50* | *GNA11* | 45 |
| ***ALK*** | ***434*** | *GNAQ* | 32 |
| *APC* | *675* | *GNAS* | 90 |
| *AR* | *126* | *HRAS* | 105 |
| *ARAF* | *66* | *IDH1* | 43 |
| ***BRAF*** | ***150*** | *IDH2* | 101 |
| *CCND1* | *267* | ***KIT*** | 609 |
| *CCND2* | *410* | *KRAS* | 188 |
| *CCND3* | *537* | ***MAP2K1*** | 198 |
| *CDK4* | *388* | ***MAP2K2*** | 127 |
| *CDK6* | *351* | ***MET*** | 682 |
| *CTNNB1* | *67* | *MTOR* | 522 |
| *CHEK2* | *81* | *MYC* | 246 |
| ***DDR2*** | ***141*** | ***NRAS*** | 186 |
| ***EGFR*** | ***910*** | *NTRK1* | 124 |
| *ERBB2* | *743* | *NTRK3* | 60 |
| *ERBB3* | *265* | *PDGFRA* | 492 |
| *ERG* | *only fusions* | ***PIK3CA*** | 409 |
| *ESR1* | *144* | *PTEN* | 343 |
| *ETV1* | *only fusions* | *RAF1* | 35 |
| *FBXW7* | *290* | *RET* | 392 |
| *FGFR1* | *510* | *ROS1* | 243 |
| ***FGFR2*** | ***778*** | *SF3B1* | 35 |
| *FGFR3* | *590* | *SMAD4* | 229 |
| *FGFR4* | *96* | *SMO* | 423 |
| *FLT3* | *39* | *TP53* | 979 |

**Table S2**. Genes included in the NGS panel used. Gene symbols in bold letter have been described to be potentially relevant for ALK resistance according to Gainor et al. (1)

1. Gainor JF, Dardaei L, Yoda S, Friboulet L, Leshchiner I, Katayama R, et al. (2016) Molecular mechanisms of resistance to first- and second-generation ALK inhibitors in ALK -rearranged lung cancer. Cancer Discov 6, 1118-1133.

| **Patient** | **Sample** | **Gene** | **Amino Acid Change** | **Nucleotide Change / CytoBand** | **Digital PCR** | **Sanger** |
| --- | --- | --- | --- | --- | --- | --- |
| Patient 2 | Sample 2 | *TP53* | p.A88Rfs | c.261_262insC |  | Negative |
| Patient 2 | Sample 2 | *TP53* | p.M133T | c.398T>C |  | Negative |
| Patient 2 | Sample 2 | *TP53* | p.P92A | c.274C>G | Negative |  |
| Patient 6 | Sample 8 | *TP53* | p.P92A | c.274C>G | Negative |  |
| Patient 6 | Sample 8 | *TP53* | p.R282W | c.844C>T | Negative |  |
| Patient 7 | Sample 9 | *TP53* | p.V157F | c.469G>T |  | Negative |
| Patient 7 | Sample 9 | *TP53* | p.P92A | c.274C>G | Negative |  |
| Patient 8 | Sample 10 | *TP53* | p.P92A | c.274C>G | Negative |  |
| Patient 9 | Sample 11 | *TP53* | p.G105C | c.313G>T |  | Negative |
| Patient 10 | Sample 12 | *TP53* | p.P92A | c.274C>G | Negative |  |
| Patient 16 | Sample 18 | *TP53* | p.R158C | c.472C>T |  | Negative |
| Patient 16 | Sample 18 | *TP53* | p.P250L | c.749C>T |  | Negative |
| Patient 16 | Sample 18 | *TP53* | p.? | c.919+10C>T |  | Negative |
| Patient 16 | Sample 18 | *TP53* | p.P92A | c.274C>G | Negative |  |
| Patient 20 | Sample 24 | *TP53* | p.C176Y | c.527G>A |  | Negative |
| Patient 20 | Sample 24 | *TP53* | p.S241A | c.721T>G |  | Negative |
| Patient 20 | Sample 24 | *TP53* | p.P92A | c.274C>G | Negative |  |
| Patient 21 | Sample 25 | *TP53* | p.Q16Rfs | c.45delT |  | Negative |
| Patient 22 | Sample 26 | *TP53* | p.P92A | c.274C>G | Negative |  |

**Table S3.** DNA genotyping of PBMCs. Mutations in *TP53* gene detected in ctDNA were interrogated in the DNA from PBMCs. The table shows the methodology by which each mutation was tested. The results discard a clonal hematopoiesis origin of the mutations

Sanger sequencing LOD has been established at 20% according to manufacturer

| **cDNA** | **protein** | **transcript** | **chromosome** | **position** |
| --- | --- | --- | --- | --- |
| c.3599C>T | p.A1200V | NM_004304.4 | chr2 | 29443611 |
| c.3806G>C | p.G1269A | NM_004304.4 | chr2 | 29432682 |
| c.3605_3606delGAinsAG | p.G1202E | NM_004304.4 | chr2 | 29443611 |
| c.3821C>T | p.A1274V | NM_004304.4 | chr2 | 29432664 |
| c.3824G>A | p.R1275Q | NM_004304.4 | chr2 | 29432664 |
| c.3604G>A | p.G1202R | NM_004304.4 | chr2 | 29443611 |
| c.3455T>C | p.L1152P | NM_004304.4 | chr2 | 29445270 |
| c.3626G>A | p.R1209Q | NM_004304.4 | chr2 | 29443591 |
| c.3606A>G | p.G1202G | NM_004304.4 | chr2 | 29443611 |
| c.3586C>A | p.L1196M | NM_004304.4 | chr2 | 29443631 |
| c.3691C>T | p.R1231W | NM_004304.4 | chr2 | 29436902 |
| c.3521T>C | p.F1174S | NM_004304.4 | chr2 | 29443696 |
| c.3367G>T | p.G1123C | NM_004304.4 | chr2 | 29445466 |
| c.3600G>A | p.A1200A | NM_004304.4 | chr2 | 29443611 |
| c.3600G>T | p.A1200A | NM_004304.4 | chr2 | 29443611 |
| c.3617C>A | p.S1206Y | NM_004304.4 | chr2 | 29443600 |
| c.3601G>A | p.G1201R | NM_004304.4 | chr2 | 29443611 |
| c.3807A>G | p.G1269G | NM_004304.4 | chr2 | 29432681 |
| c.3538G>C | p.V1180L | NM_004304.4 | chr2 | 29443679 |

**Table S4.** List of mutations in *ALK* locus tested by dPCR

| ***ALK*-TKI** | **Number of samples** | ***ALK*-TKI naive (N)** | **Treatment after crizotinib (N)** |
| --- | --- | --- | --- |
| Crizotinib | 11 | 11 | - |
| Alectinib | 10 | 2 | 7 |
| Ceritinib | 4 | 0 | 4 |
| Lorlatinib | 2 | 0 | 0 |
| Brigatinib | 1 | 0 | 1 |

**Table S5. *ALK*-Is treatments of the study cohort.** Number of samples collected upon progression and according to *ALK*-TKIs. Number of samples collected upon progression to a first line treatment with an *ALK*-Is (*ALK*-TKI naive). Number of samples that correspond to patients who were previously treated with a previous line of treatment with crizotinib.

| **Sample** | **Gene (HUGO Symbol)** | **Amino Acid Change** | **Nucleotide Change / CytoBand** | **MAF NGS** | **Type of mutation** | **Variant Class (Tier)** | **Transcript** | **rs** | **COSM** |
| --- | --- | --- | --- | --- | --- | --- | --- | --- | --- |
| Sample 1 | *CCND3* | p.T191T | c.573A>T | 0.93 | SNV | Unknown | NM_001760 | - | - |
| Sample 1 | *TP53* | p.P92A | c.274C>G | 0.30 | SNV | unknown | NM_000546.5 | - | COSM45844 |
| Sample 2 | *ALK* | p.A1200V | c.3599C>T | 0.02 | SNV | Strong Clinical Significance | NM_004304.4 | [rs200585833](https://www.ncbi.nlm.nih.gov/snp/rs200585833) | COSM317003 |
| Sample 2 | *TP53* | p.A88Rfs | c.261_262insC | 0.52 | InDel | unknown | NM_000546.5 | - | - |
| Sample 2 | *TP53* | p.M133T | c.398T>C | 0.55 | SNV | unknown | NM_000546.5 | rs28934873 | COSM43723 |
| Sample 2 | *TP53* | p.P92A | c.274C>G | 0.30 | SNV | unknown | NM_000546.5 | - | COSM45844 |
| Sample 3 | *ALK* | p.G1269A | c.3806G>C | 3.36 | SNV | Strong Clinical Significance | NM_004304.4 | [rs1057519781](https://www.ncbi.nlm.nih.gov/variation/tools/1000genomes/?chr=2&from=29432682&to=29432682&gts=rs1057519781&mk=29432682:29432682\|rs1057519781) | COSM1169707 |
| Sample 3 | *TP53* | p.L35fs | c.104_105insC | 0.30 | InDel | unknown | NM_000546.5 | - | - |
| Sample 4 | *BRAF* | p.G466V | c.1397G>T | 0.49 | SNV | Potential Clinical Significance | NM_004333.4 | rs121913351 | COSM451 |
| Sample 4 | *TP53* | p.G262Rfs | c.784insC | 1.00 | InDel | unknown | NM_000546.5 | - | - |
| Sample 4 | *TP53* | p.V197Gfs | c.590delT | 0.44 | InDel | unknown | NM_000546.5 | - | COSM44655 |
| Sample 6 | *ALK* | p.G1269A | c.3806G>C | 0.88 | SNV | Strong Clinical Significance | NM_004304.4 | [rs1057519781](https://www.ncbi.nlm.nih.gov/variation/tools/1000genomes/?chr=2&from=29432682&to=29432682&gts=rs1057519781&mk=29432682:29432682\|rs1057519781) | COSM1169707 |
| Sample 7 | *ALK* | p.G1202R | c.3604G>A | 1.28 | SNV | Strong Clinical Significance | NM_004304.4 | rs1057519783 | COSM144250 |
| Sample 7 | *TP53* | p.P92A | c.274C>G | 0.90 | SNV | unknown | NM_000546.5 | - | COSM45844 |
| Sample 8 | *ALK* | p.L1196M | c.3586C>A | 0.02 | SNV | Strong Clinical Significance | NM_004304.4 | rs1057519784 | COSM99137 |
| Sample 8 | *FGFR2* | p.G305R | c.913G>A | 0.05 | SNV | Unknown | [NM_000141.5](http://www.ncbi.nlm.nih.gov/nuccore/NM_000141.5) | - | COSM29824 |
| Sample 8 | *TP53* | p.P92A | c.274C>G | 0.18 | SNV | unknown | NM_000546.5 | - | COSM45844 |
| Sample 8 | *TP53* | p.R282W | c.844C>T | 0.17 | SNV | unknown | NM_000546.5 | rs28934574 | COSM10704 |
| Sample 9 | *SMAD4* | p.A118V | c.353C>T | 0.03 | SNV | Unknown | NM_005359.6 | - | COSM14215 |
| Sample 9 | *TP53* | p.P92A | c.274C>G | 0.84 | SNV | unknown | NM_000546.5 | - | COSM45844 |
| Sample 9 | *TP53* | p.V157F | c.469G>T | 0.99 | SNV | unknown | NM_000546.5 | rs121912654 | COSM10670 |
| Sample 10 | *IDH2* | p.R140Q | c.419G>A | 0.06 | SNV | Potential Clinical Significance | NM_002168.3 | - | COSM41590 |
| Sample 10 | *TP53* | p.P92A | c.274C>G | 0.84 | SNV | unknown | NM_000546.5 | - | COSM45844 |
| Sample 11 | *MET* | p.L7P | c.20T>C | 0.43 | SNV | Unknown | NM_001127500 | - | - |
| Sample 11 | *TP53* | p.G105C | c.313G>T | 0.42 | SNV | unknown | NM_000546.5 | - | COSM44481 |
| Sample 12 | *MAP2K1* | p.F129L | c.385T>C | 1.00 | SNV | Potential Clinical Significance | NM_002755.3 | rs1057519805 | COSM1235480 |
| Sample 12 | *TP53* | p.P92A | c.274C>G | 1.37 | SNV | unknown | NM_000546.5 | - | COSM45844 |
| Sample 13 | *TP53* | p.A88Rfs | c.261_262insC | 0.66 | InDel | unknown | NM_000546.5 | - | - |
| Sample 13 | *TP53* | p.P92A | c.274C>G | 0.45 | SNV | unknown | NM_000546.5 | - | COSM45844 |
| Sample 14 | *CCND1* |  | Loss (0,41) |  | CNV | Unknown | NM_053056 | - | - |
| Sample 14 | *FGFR3* |  | Loss (0,38) |  | CNV | Unknown | NM_000142 | - | - |
| Sample 14 | *MYC* |  | Gain (3,08) |  | CNV | Potential Clinical Significance | NM_005359.5 | - | - |
| Sample 14 | *TP53* | p.M160I | c.480G>T | 0.75 | SNV | unknown | NM_000546.5 | [rs772354334](https://www.ncbi.nlm.nih.gov/snp/rs772354334) | COSM45674 |
| Sample 16 | *PIK3CA* | p.E545K | c.1633G>A | 0.76 | SNV | Potential Clinical Significance | NM_006218 | rs104886003 | COSM763 |
| Sample 18 | *ALK* | p.G1202R | c.3604G>A | 0.04 | SNV | Strong Clinical Significance | NM_004304.4 | rs1057519783 | COSM144250 |
| Sample 18 | *ALK* | p.S1206Y | c.3617C>A | 0.06 | SNV | Strong Clinical Significance | NM_004304.4 | [rs1057519782](https://www.ncbi.nlm.nih.gov/variation/tools/1000genomes/?chr=2&from=29443600&to=29443600&gts=rs1057519782&mk=29443600:29443600\|rs1057519782) | COSM144251 |
| Sample 18 | *TP53* | p.? | c.919+10C>T | 0.09 | SNV | unknown | NM_000546.5 | - | COSM45540 |
| Sample 18 | *TP53* | p.P250L | c.749C>T | 0.08 | SNV | unknown | NM_000546.5 | rs1064794311 | COSM10771 |
| Sample 18 | *TP53* | p.P92A | c.274C>G | 0.18 | SNV | unknown | NM_000546.5 | - | COSM45844 |
| Sample 18 | *TP53* | p.R158C | c.472C>T | 0.51 | SNV | unknown | NM_000546.5 | rs587780068 | COSM43848 |
| Sample 19 | *TP53* | p.R273G | c.817C>G | 0.64 | SNV | unknown | NM_000546.5 | [rs121913343](https://varsome.com/variant/hg19/rs121913343) | COSM43843 |
| Sample 20 | *PIK3CA* | p.E545A | c.1634A>C | 0.79 | SNV | Potential Clinical Significance | NM_006218 | rs121913274 | COSM12458 |
| Sample 20 | *TP53* | p.S149Pfs | c.445delT | 0.85 | InDel | unknown | NM_000546.5 | rs1064793929 | COSM44099 |
| Sample 21 | *EGFR* | p.E746_A750del | c.2235_2249delGGAATTAAGAGAAGC | 1.63 | InDel | Strong Clinical Significance | NM_005228.4 | - | COSM6223 |
| Sample 21 | *TP53* | p.R282W | c.844C>T | 5.09 | SNV | unknown | NM_000546.5 | rs28934574 | COSM10704 |
| Sample 23 | *ALK* | p.L1196M | c.3586C>A | 0.05 | SNV | Strong Clinical Significance | NM_004304.4 | rs1057519784 | COSM99137 |
| Sample 23 | *ALK* | p.R1275Q | c.3824G>A | 0.03 | SNV | Strong Clinical Significance | NM_004304.4 | rs113994087 | COSM28056 |
| Sample 23 | *TP53* | p.E221fs | c.662delA | 0.10 | InDel | unknown | NM_000546.5 | - | COSM85959 |
| Sample 23 | *TP53* | p.I251fs | c.751_752insC | 0.21 | InDel | unknown | NM_000546.5 | - | - |
| Sample 24 | *ALK* | p.G1202R | c.3604G>A | 0.05 | SNV | Strong Clinical Significance | NM_004304.4 | rs1057519783 | COSM144250 |
| Sample 24 | *TP53* | p.C176Y | c.527G>A | 0.15 | SNV | unknown | NM_000546.5 | rs786202962 | COSM10687 |
| Sample 24 | *TP53* | p.P92A | c.274C>G | 0.18 | SNV | unknown | NM_000546.5 | - | COSM45844 |
| Sample 24 | *TP53* | p.S241A | c.721T>G | 0.09 | SNV | unknown | NM_000546.5 | rs1057520002 | COSM44224 |
| Sample 25 | *PIK3CA* | p.I338F | c.1012A>T | 0.32 | SNV | Unknown | NM_006218 | - | - |
| Sample 25 | *TP53* | p.Q16Rfs | c.45delT | 1.57 | InDel | unknown | NM_000546.5 | [rs1555526997](https://varsome.com/variant/hg19/rs1555526997) | COSM46341 |
| Sample 26 | *ALK* | p.G1202R | c.3604G>A | 0.03 | SNV | Strong Clinical Significance | NM_004304.4 | rs1057519783 | COSM144250 |
| Sample 26 | *TP53* | p.P92A | c.274C>G | 1.23 | SNV | unknown | NM_000546.5 | - | COSM45844 |
| Sample 27 | *MAP2K1* | p.F129L | c.385T>C | 0.06 | SNV | Potential Clinical Significance | NM_002755.3 | rs1057519805 | COSM1235480 |
| Sample 27 | *SMAD4* | p.R361C | c.1081C>T | 0.03 | SNV | Unknown | NM_005359.5 | rs80338963 | COSM14140 |
| Sample 27 | *TP53* | p.P92A | c.274C>G | 0.26 | SNV | unknown | NM_000546.5 | - | COSM45844 |
| Sample 28 | *ALK* | p.V1180L | c.3538G>C | 0.37 | SNV | Strong Clinical Significance | NM_004304.4 | - | COSM4381101 |

**Table S6.** List of all somatic mutations detected in the study cohort.

| ***ALK* cohort** | | | | | |  |
| --- | --- | --- | --- | --- | --- | --- |
|  |  | dPCR | | | |  |
|  |  | + | | - | |  |
| V*ALK* TOOL | + | 8 | | 2 | |  |
|  | - | 4 | | 27 | |  |
|  |  | |  | |  |  |
| PPA (%) | 66.67 | |  | |  |  |
| NPA (%) | 93.10 | |  | |  |  |
| ORA (%) | 85.37 | |  | |  |  |
|  |  | |  | |  |  |

**Table S7**. Cross-table describing PPA, NPA and ORA for *ALK* cohort using V*ALK* tool

| **Valencia cohort** | | | | | |
| --- | --- | --- | --- | --- | --- |
|  |  | dPCR | | | |
|  |  | + | | | - |
| V*ALK* TOOL | + | 3 | | | 1 |
|  | - | 0 | | | 51 |
|  |  | |  |  | |
| PPA (%) | 100.00 | |  |  | |
| NPA (%) | 98.08 | |  |  | |
| ORA (%) | 98.18 | |  |  | |

**Table S8**. Cross-tables describing PPA, NPA and ORA for Valencia cohort using V*ALK* tool

| ***ALK* cohort** | | | | |  |  |
| --- | --- | --- | --- | --- | --- | --- |
|  |  | dPCR | | |  |  |
|  |  | + | | - |  |  |
| Oncomine Filter | + | 3 | | 1 |  |  |
|  | - | 9 | | 27 |  |  |
|  |  | |  | | |  |
| PPA (%) | 25.00 | |  | | |  |
| NPA (%) | 96.43 | |  | | |  |
| ORA (%) | 75.00 | |  | | |  |

**Table S9**. Cross-tables describing PPA, NPA and ORA for *ALK* cohort using the Oncomine Filter

| **Valencia cohort** | | | | |  |  |
| --- | --- | --- | --- | --- | --- | --- |
|  |  | dPCR | | |  |  |
|  |  | + | | - |  |  |
| Oncomine Filter | + | 1 | | 0 |  |  |
|  | - | 2 | | 51 |  |  |
|  |  | |  | | |  |
| PPA (%) | 33.33 | |  | | |  |
| NPA (%) | 100.00 | |  | | |  |
| ORA (%) | 96.30 | |  | | |  |

**Table S10.** Cross-tables describing PPA, NPA and ORA for Valencia cohort using the Oncomine Filter

| **Nº Patient** | **Nº Sample** | **Mutation** | **Method used in progression sample** | **Previous sample** | **Method used in previous sample** | **Result** |
| --- | --- | --- | --- | --- | --- | --- |
| Patient 3 | Sample 4 | *BRAF*; p.G466V/c.1397G>T | NGS | PLASMA | NGS | Absence |
| Patient 5 | Sample 6 | *ALK*; p.G1269A/c.3806G>C | NGS + dPCR | PLASMA | dPCR | Absence |
| Patient 5 | Sample 7 | *ALK*; p.G1202R/c.3604G>A | NGS + dPCR | PLASMA | NGS + dPCR | Absence |
| Patient 10 | Sample 12 | *MAP2K1*; p.F129L/c.385T>C | NGS | PLASMA + TUMOR | NGS | Absence |
| Patient 14 | Sample 16 | *PIK3CA*; p.E545K/c.1633G>A | NGS + dPCR | PLASMA | dPCR | Absence |
| Patient 18 | Sample 20 | *PIK3CA*; p.E545A/c.1634A>C | NGS | TUMOR | dPCR | Absence |
| Patient 19 | Sample 23 | *ALK*; p.L1196M/c.3586C>A | NGS | PLASMA | NGS | Absence |
| Patient 19 | Sample 23 | *ALK*; p.R1275Q /c.3824G>A | NGS | PLASMA | NGS | Absence |

**Table S11.** List of the mutations detected at disease progression and status at baseline or in previous sample.
